# Influence of disease prevalence on cost-effectiveness of magnetic resonance imaging versus coronary angiography combined with fractional flow reserve testing

**DOI:** 10.1186/1532-429X-16-S1-O98

**Published:** 2014-01-16

**Authors:** Karine Moschetti, David Favre, Christophe Pinget, Guenter Pilz, Steffen E Petersen, Anja Wagner, Jean-Blaise Wasserfallen, Juerg Schwitter

**Affiliations:** 1 IEMS and UET, University of Lausanne (Unil) and University Hospital (CHUV), Lausanne, Switzerland; 2 IEMS, University of Lausanne, Lausanne, Switzerland; 3 UET, University Hospital (CHUV), Lausanne, Switzerland; 4 Klinik Agatharied, Akademisches Lehrkrankenhaus der LMU, Munich, Hausham, Germany; 5 National Institute for Health Research Cardiovascular Biomedical Research Unit at Barts, Queen Mary University of London, London, UK; 6 Comprehensive Cardiology of Stamford and Greenwich, Stamford, CT 06902, Connecticut, USA; 7 IEMS and UET, University of Lausanne (Unil) and University Hospital (CHUV), Lausanne, Switzerland; 8 Divison of Cardiology and Cardiac MR Center, University Hospital (CHUV), Lausanne, Switzerland

## Background

According guidelines, patients with coronary artery disease (CAD) should undergo revascularization if myocardial ischemia is present. While coronary angiography (CXA) allows the morphological assessment of CAD, the fractional flow reserve (FFR) has proved to be a complementary invasive test to assess the functional severity of CAD, i.e. to detect ischemia. Perfusion Cardiac Magnetic Resonance (CMR) has emerged as a robust non-invasive technique to assess myocardial ischemia. The goal of the study was to compare the cost-effectiveness ratio - defined as the costs per patient correctly diagnosed - of two algorithms used to diagnose hemodynamically significant CAD in relation to the pretest likelihood of CAD: 1) a CMR to assess ischemia before referring positive patients to CXA (CMR+CXA), 2) a CXA in all patients combined with a FFR test in patients with angiographically positive stenoses (CXA+FFR).

## Methods

The costs, evaluated from the health care system perspective in the Swiss, German, United Kingdom (UK), and the United States (US) contexts, included public prices of the different tests considered as outpatient procedures, complications' costs and costs induced by diagnosis errors (false negative). The effectiveness criterion was the ability to accurately identify a patient with significant, i.e. hemodynamically relevant CAD. Test performances used in the model were based on clinical literature. Using a mathematical model, we compared the cost-effectiveness ratio for both algorithms for hypothetical patient cohorts with different pretest likelihood of CAD.

## Results

The cost-effectiveness ratio decreased hyperbolically with increasing pretest likelihood of CAD for both strategies. CMR+CXA and CXA+FFR were equally cost-effective at a pretest likelihood of CAD of 62% in Switzerland, 65% in Germany, 83% in the UK, and 84% in the US with costs of CHF 5'793, € 1'517, £ 2'683, and $ 2'128 per patient correctly diagnosed. Below these thresholds, CMR+CXA showed lower costs per patient correctly diagnosed than CXA+FFR.

## Conclusions

Implications for the health care system/professionals/patients/society The CMR+CXA strategy is more cost-effective than CXA+FFR below a CAD prevalence of 62%, 65%, 83%, and 84% for the Swiss, the German, the UK, and the US health care systems, respectively. These findings may help to optimize resource utilization in the diagnosis of coronary artery disease. They show to what extent the cost-effectiveness to diagnose CAD depends on the prevalence of the disease.

## Funding

No.

